# Effect of drought stress on neutral detergent fiber degradation kinetics of corn for silage*

**DOI:** 10.3168/jdsc.2022-0326

**Published:** 2023-02-02

**Authors:** Gonzalo Ferreira, Christy L. Teets, Anthony M. Kingori, James O. Ondiek

**Affiliations:** 1 School of Animal Sciences, Virginia Polytechnic Institute and State University, Blacksburg 24061; 2 Department of Animal Science, Egerton University, Njoro, Kenya, 20115

## Abstract

The objective of this study was to determine the effect of irrigation on in situ neutral detergent fiber (NDF) degradability of corn tissues from plants grown under controlled conditions in a greenhouse. Five commercial corn hybrids were planted in 6 pots that were placed in a greenhouse. Pots were randomly subjected to 2 irrigation regimens, which consisted of either abundant (A; 598 mm) or restricted (R; 273 mm) irrigation. At harvesting, leaf blades and stem internodes were collected from the upper and bottom portion of the plants. Tissue samples were incubated in the rumen of 3 rumen-cannulated cows for 0, 3, 6, 12, 24, 48, 96, and 240 h to determine in situ NDF degradation kinetics. Drought stress did not affect the concentration of undegraded NDF (uNDF) in upper or bottom internodes but slightly decreased it in upper leaf blades (17.5 and 15.7% for A and R, respectively). The concentration of uNDF differed substantially among corn hybrids in upper internodes (13.4 to 28.3% uNDF), bottom internodes (21.5 to 42.3% uNDF), and upper leaf blades (11.6 to 20.1% uNDF). No interactions existed between irrigation treatment and corn hybrid for uNDF concentration. Drought stress did not affect the fractional degradation rate (*kd*) of NDF in upper internodes, bottom internodes, or upper leaf blades. The *kd* of NDF differed among corn hybrids in upper (3.8 to 6.6%/h) and bottom internodes (4.2 to 6.7%/h) but did not vary in upper leaf blades (3.8%/h). No interactions existed between irrigation treatment and corn hybrids for the *kd* of NDF. Significant interactions existed between irrigation treatment and corn hybrids for the effective ruminal degradation (ERD) of NDF in upper and bottom internodes. This interaction did not exist for upper leaf blades. The ERD of NDF differed substantially among corn hybrids in upper leaf blades (32.5 to 39.1%). In conclusion, drought-stressed corn had a marginal increase of NDF degradability of leaf blades but not in stem internodes, and drought stress had no effects on ERD of NDF. The effect of drought stress on NDF degradability of corn for silage is still inconclusive and deserves further investigation.

## Highlights

• Drought stress affects NDF degradability marginally.
• Drought stress had no effects on the effective ruminal degradation of NDF.
• The effect of drought stress on NDF degradability of corn for silage is still inconclusive.

## Graphical Abstract

**Figure:**
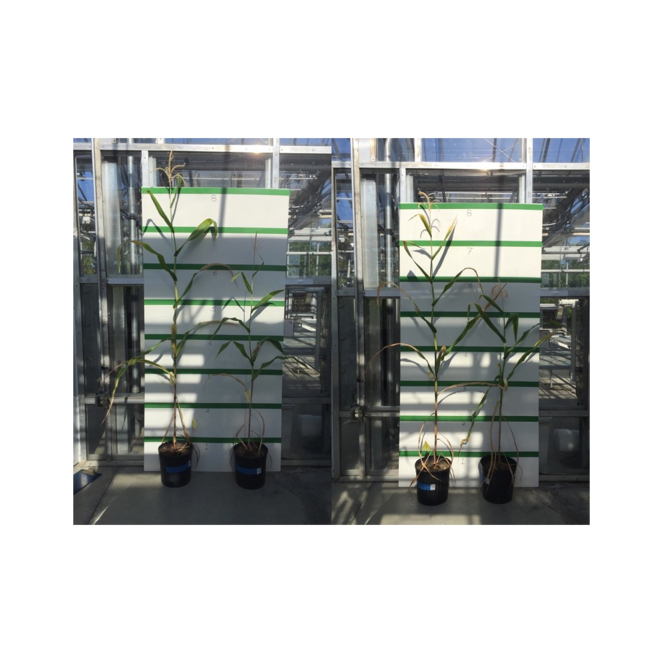
**Summary:** Limited information exists about the effect of drought stress on forage fiber digestibility. In a greenhouse, we evaluated the effect of drought stress on neutral detergent fiber (NDF) digestibility of corn tissues. Corn plants were subjected to abundant (598 mm) or restricted (273 mm) irrigation regimens, and stems and leaves were incubated in the rumen of dairy cows. According to the conditions of this study, we concluded that drought-stressed corn had a marginal increase in NDF degradability of leaf blades but not in stem internodes, that drought stress had no effects on the effective ruminal degradation of NDF when comparing irrigation treatments within hybrids, and that the effect of drought stress on NDF degradability of corn for silage is still inconclusive. We also observed that the growth of corn plants in a greenhouse does not result in plants with the anatomical structure typically seen in the field.

Drought stress has a tremendous negative impact on the agricultural economy and global food security (Brown and Funk, 2008; Farooq et al., 2009). According to the United Nations Office for Disaster Risk Reduction (United Nations, 2021), the droughts of 2008–2009 in Argentina and 2017 in the United States resulted in agricultural losses of $4.0 and $2.6 billion, respectively. Even worse, according to the National Center of Environmental Information (National Oceanic and Atmospheric Administration), the droughts from 1988 and 2012 in the United States resulted in agricultural losses ranging from $30 to $40 billion per event (Rippey, 2015).

Drought is defined as an exceptional lack of water compared with normal conditions (Van Loon et al., 2016). From an agricultural perspective, the major and most direct effect of drought stress is on the yield reduction of crops for grains and forages and yield reduction of pastures and rangelands. At a plant tissue level, however, limited information exists regarding the impact of drought stress on forage quality (Lohölter et al., 2012; Ferreira et al., 2015, 2021a,b). Even more, the current understanding of the impact of drought stress on corn silage degradability is confusing. In the United States, for example, a belief exists that water stress increases NDF degradability of corn silage (Mahana and Thomas, 2011; Hutjens, 2017; Ferreira, 2020). However, one of the few controlled studies addressing this possibility does not support such a belief (Ferreira et al., 2021b). Specifically, Ferreira et al. (2021b) reported that drought-stressed corn had a lower in vitro NDF degradability in corn internodes than non-drought-stressed corn, although that effect did not exist in corn leaf blades. The latter observation suggested that the induced drought might not have been strong enough to exacerbate the effects of drought stress on NDF degradability.

Given the difficulties associated with controlling environmental conditions (Farooq et al., 2009), controlled studies comparing the nutritional quality of drought-stressed and non-drought-stressed corn for silage are limited (Lohölter et al., 2012; Ferreira et al., 2021a,b). In addition to limited environment control, the limited replication and randomization of experimental units (i.e., plots) in drought stress studies limits the interpretation and utility of the data. To address those limitations, Ferreira et al. (2021a,b) performed a controlled irrigation study considering randomization and replication of treatments, although the flooding irrigation method used may have restrained the possibility of inducing severe drought stress.

The hypothesis of this study is that drought stress does not increase NDF degradability of corn for silage, and we performed a greenhouse study to better control the environment and test this hypothesis. Hence, the objective of this study was to determine the effect of irrigation on in situ NDF degradability of corn tissues from plants grown under controlled conditions in a greenhouse.

Five commercial corn hybrids (3 conventional and 2 brown midrib) were planted in mini-pots at the Dairy Nutrition Laboratory at Virginia Tech. After emergence, 6 mini-pots per hybrid were transferred to 6 pots that were later placed in a greenhouse. Pots were randomly subjected to 2 irrigation regimens, which consisted of either 598 or 273 mm of water (Figure 1) for abundant (**A**) and restricted (**R**) irrigation, respectively. An irrigation regimen of 600 mm during the growing season was considered abundant based on Ferreira et al. (2014) and Xue et al. (2017). Arbitrarily, we considered 50% irrigation sufficient to induce drought stress.

**Figure 1:**
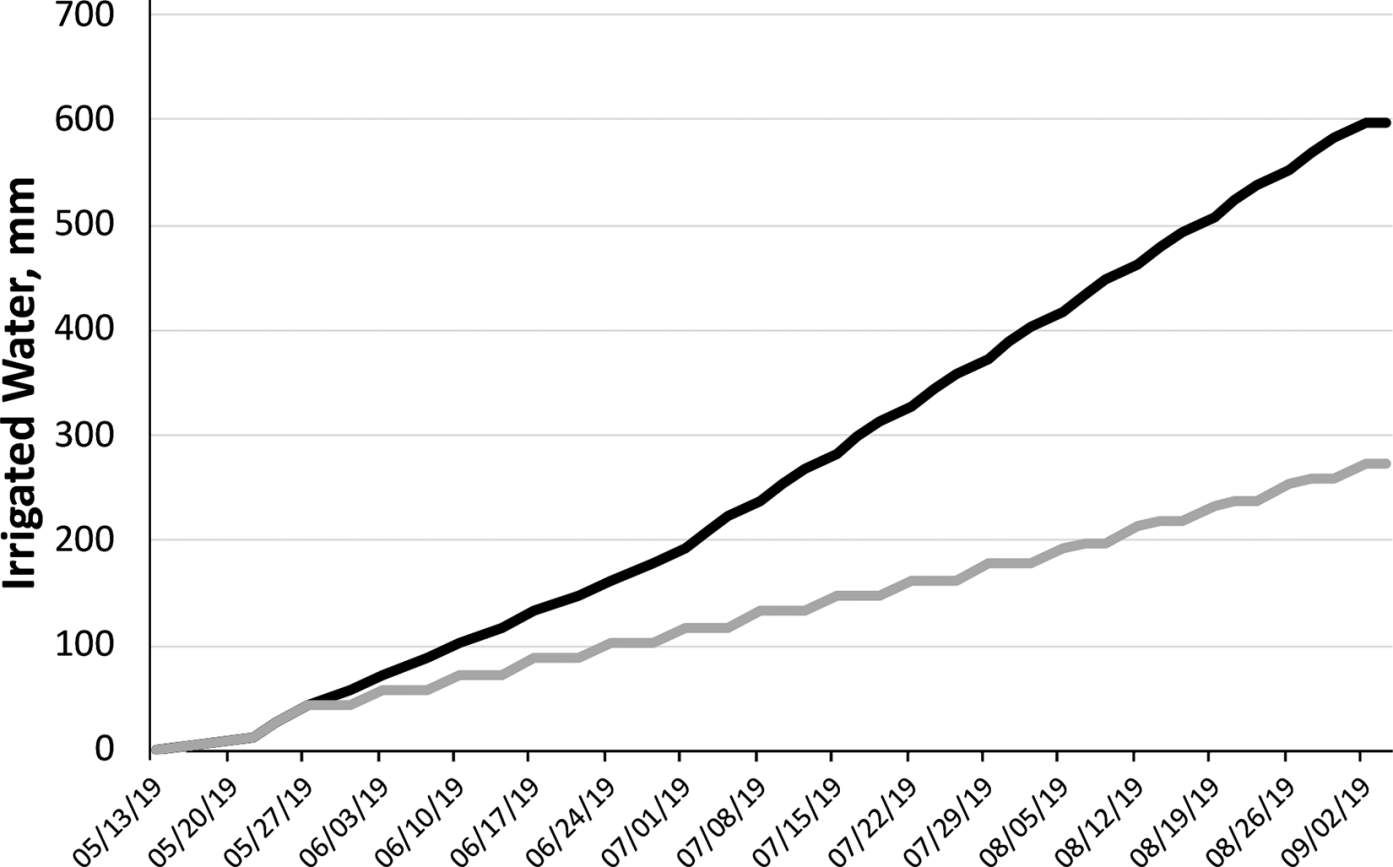
Cumulative irrigated water of corn plants. Control plants (black) received abundant (598 mm) irrigated water, whereas drought-stressed plants (gray) received restricted (273 mm) irrigated water.

At harvesting, which occurred on a single day and when hybrids showed early-dent to 1/4-milkline stage of maturity, leaf blades and stem internodes were collected from the upper and bottom portion of the plants. Tissue samples were dried at 55°C for 48 h and ground to pass through a 1-mm screen of a Wiley mill. Ground samples (0.25 g) were inserted into acetone-rinsed porous bags (F57, Ankom Technology) and incubated in the rumen of 3 rumen-cannulated cows fed a TMR containing 32% corn silage, 3% alfalfa hay, and 65% concentrate mix (DM basis). Bags were extracted from the rumen 0, 3, 6, 12, 24, 48, 96, and 240 h after initial incubation, rinsed 3 times (3-min washing cycles) using a washing machine (SKY2767, Best Choice Products), and dried in a forced-air oven at 55°C for 48 h. All procedures involving animals were approved by the Institutional Animal Care and Use Committee of Virginia Tech (Protocol 18–229).

The concentration of NDF was determined in forage tissues and forage tissue residues after in situ ruminal fermentation. Ash-free NDF was determined using the Ankom200 Fiber Analyzer (Ankom Technology). Sodium sulfite and α-amylase (Ankom Technology) were included for NDF analysis (Ferreira and Mertens, 2007; Ferreira and Thiex, 2023).

Degradation kinetic parameters (Ferreira et al., 2022) were estimated using the NLIN procedure of SAS (SAS version 9.4, SAS Institute Inc.) and according to the in situ NDF degradability model = {(100 – uNDF) × [1 – *e*^(-^*^kd^* ^× T)^]}, where T is the time of fermentation in hours, uNDF is the undigested NDF (**uNDF**) as a percent of initial NDF after 240 h of fermentation, and *kd* is the fractional disappearance rate per hour of the potentially degradable NDF (**pdNDF**). The effective ruminal degradability (**ERD**) of pdNDF (% NDF) was determined as ERD = pdNDF × *kd*/(*kd*+*kp*), where *kp* is the passage rate considered at 5%/h (Kammes and Allen, 2012).

The experiment was designed and analyzed as a randomized complete block design with a 2 × 5 factorial arrangement of treatments with 3 replicates, where irrigation (A vs R) and hybrids (1 to 5) were the experimental factors. The model included the effects of cow (random; 2 df), irrigation (fixed; 1 df), hybrid (fixed; 4 df), the irrigation by hybrid interaction (fixed; 4 df), and the random residual error (18 df). Statistical differences between main effects and statistical interactions between factors were declared at *P* < 0.05 and *P* < 0.10, respectively. Protected multiple comparisons were performed according to the method of Tukey when statistical interactions existed.

Corn plants subjected to limited irrigation showed clear signs of drought stress, such as folded leaf blades and shorter stem internodes that resulted in shorter plants (Figure 2). Due to the limited amount of tissue harvested, we did not analyze data for the bottom leaf blades (Table 1).

**Figure 2:**
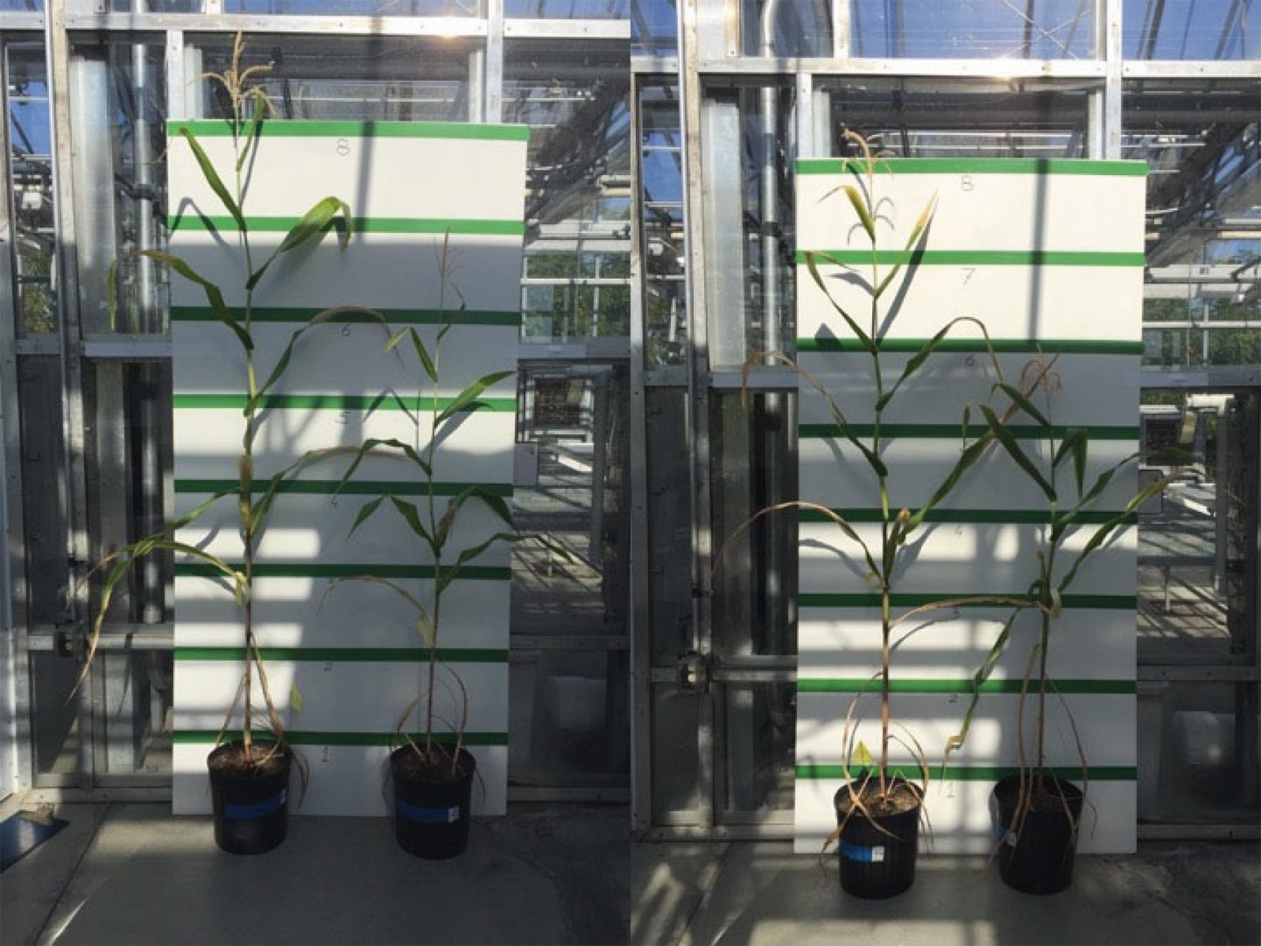
Effect of drought stress on corn plant growth. Plants were grown in a greenhouse under controlled irrigation regimens. Each of the 2 pictures depicts corn plants grown with either abundant water (598 mm; left) or limited (273 mm, right) water.

**Table 1:** In situ ruminal fiber degradation kinetics of 5 corn hybrids (H1 to H5) grown in a greenhouse at 2 irrigation regimens [600 and 300 mm of water for abundant (A) and restricted (R) irrigation, respectively]

Item^1^	H1		H2		H3		H4		H5		SEM	*P*<^2^		
	A	R	A	R	A	R	A	R	A	R		I	H	I × H
Upper internodes														
pdNDF, % NDF	76.0	75.4	72.1	73.1	86.1	87.0	71.4	72.0	72.7	76.7	2.1	0.31	0.01	0.76
uNDF, % NDF	24.0	24.6	27.9	26.9	13.9	13.0	28.6	28.0	17.3	23.3	2.1	0.31	0.01	0.76
*kd*, %/h	3.65^b^	4.23^b^	3.25^b^	4.31^b^	3.62^b^	5.36^ab^	7.43^a^	5.84^ab^	4.42^b^	5.99^ab^	0.7	0.10	0.01	0.07
ERD, % pdNDF	32.1^cd^	34.3abcd	28.4^d^	33.5bcd	36.1abcd	44.4^a^	42.2^ab^	38.4abc	33.8bcd	41.7abc	2.2	0.01	0.01	0.04
Bottom internodes														
pdNDF, % NDF	65.2	64.3	61.3	59.6	75.8	81.2	62.9	63.9	54.4	61.1	2.6	0.23	0.01	0.42
uNDF, % NDF	34.8	35.7	38.7	40.4	24.2	18.8	37.1	36.1	45.6	38.9	2.6	0.23	0.01	0.42
*kd*, %/h	4.59	3.70	5.73	5.98	4.33	4.59	6.61	6.77	4.15	6.65	0.8	0.35	0.02	0.29
ERD, % pdNDF	30.7^ab^	27.3^b^	32.6^ab^	31.4^ab^	35.1^ab^	38.8^a^	34.9^ab^	36.6^ab^	24.6^b^	34.8^ab^	2.2	0.13	0.01	0.05
Upper blades														
pdNDF, % NDF	79.5	80.3	82.0	83.1	88.4	88.4	78.1	82.3	84.5	87.4	1.4	0.02	0.01	0.33
uNDF, % NDF	20.5	19.7	18.0	16.9	11.6	11.6	21.9	17.7	15.5	12.6	1.4	0.02	0.01	0.33
*kd*, %/h	3.27	3.67	3.58	4.24	4.26	3.76	3.39	3.80	3.76	4.00	0.5	0.42	0.30	0.21
ERD, % pdNDF	31.6	33.7	34.1	37.3	40.4	37.8	34.4	35.2	36.0	38.6	2.7	0.14	0.01	0.23

a–d Different superscripts in the same row indicate significant differences (*P* < 0.05).

1 pdNDF = potentially digestible neutral detergent fiber; uNDF = undegraded neutral detergent fiber (after 240 h of fermentation); *kd* = fractional degradation rate of pdNDF; ERD = effective ruminal degradation.

2 I = irrigation; H = hybrid; and I × H = interaction between irrigation and hybrid.

Drought stress did not affect the concentration of uNDF in upper (*P* = 0.31) or bottom (*P* = 0.23) internodes but slightly decreased it in upper leaf blades (17.5 and 15.7% for A and R, respectively; *P* < 0.01). The concentration of uNDF differed substantially among corn hybrids in upper internodes (13.4 to 28.3% uNDF; *P* < 0.01), bottom internodes (21.5 to 42.3% uNDF; *P* < 0.01), and upper leaf blades (11.6 to 20.1% uNDF; *P* < 0.01). No interactions existed between irrigation treatment and corn hybrid for uNDF concentration.

Drought stress did not affect the *kd* of NDF in upper internodes (*P* = 0.10), bottom internodes (*P* = 0.35), or upper leaf blades (*P* = 0.42). The *kd* of NDF differed among corn hybrids in upper (3.8 to 6.6%/h) and bottom internodes (4.2 to 6.7%/h) but did not vary in upper leaf blades (3.8%/h). No interactions existed between irrigation treatment and corn hybrids for the *kd* of NDF.

Interactions existed between irrigation treatment and corn hybrids for the ERD of pdNDF in upper (*P* = 0.04) and bottom (*P* = 0.05) internodes. This interaction did not exist for upper leaf blades (*P* = 0.23). The ERD of pdNDF differed substantially among corn hybrids in upper leaf blades (32.5 to 39.1%).

Evaluating the effect of drought stress on NDF degradability has limitations. From one side, it is extremely hard to control environmental conditions to induce and manage drought stress (Farooq et al., 2009). From the other side, growing plants in controlled environments, such as water exclusion shelters or greenhouses, may affect the growing conditions in unusual ways. In this study, for example, we observed very strange looking corn plants with very thin stem internodes, even to the point of needing some assisted support (i.e., stakes) to avoid lodging.

In a recent study, Ferreira et al. (2021b) concluded that drought stress did not increase NDF degradability after seeing a marginal decrease in NDF degradability in drought-stressed corn internodes and claimed that such a result is contrary to the belief of the industry. To follow up, this study was designed to better control the growing conditions. Based on the uNDF concentration after 240 h of fermentation observed in this study, drought stress marginally increased NDF degradability in upper leaf blades but not in stem internodes. This observation is contrary to that of Ferreira et al. (2021b). Although unclear, a difference in the proportion of the midrib over the leaf blade due to the greenhouse growing conditions might explain this difference. After analyzing the interactions between irrigation and hybrid on ERD, a pattern can be observed indicating that (as a main effect) drought stress increased ERD in upper internodes, although no statistical differences between irrigation treatments were observed within the same hybrid (Table 1). The observations of the current study agree with Soderlund et al. (2012), who reported increases of NDF degradability from 50 to 55% with decreasing irrigation. However, Soderlund et al. (2012) measured NDF degradability of whole plants and not plant tissues, which may have confounded the structural composition of the plant with NDF degradability at the tissue level.

Based on this and our previous study (Ferreira et al., 2021b), drought stress has a minimal impact on NDF degradability at the tissue level. Ferreira (2020) attributed the misconception that NDF degradability is sensitive to drought stress (Mahana and Thomas, 2011; Hutjens, 2017) to the early harvesting that typically occurs under a drought stress scenario. Under this scenario, the leaves of the corn plants dry and become brownish and brittle, especially if the drought is accompanied by hot days of very low relative humidity. It is not uncommon for farmers to become anxious about losing forage quality and start harvesting the fields sooner than under normal conditions. A corn crop harvested 3 to 4 wk earlier than anticipated results in a corn crop harvested at earlier phenological stages that would have accumulated much fewer growing-degree-units (Ferreira et al., 2020) than the same crop harvested under normal conditions. This would result in less lignification of the cell wall of greater fiber degradability. A follow-up study evaluating the interaction of drought stress and the maturity at harvest of corn for silage is warranted to support our postulate (Ferreira, 2020). It is worth highlighting, however, that such a study might not be easily accomplished based on the difficulties of performing drought stress studies.

We conclude 3 things from this study. First, drought-stressed corn had a marginal increase in NDF degradability of leaf blades but not in stem internodes. Second, when comparing irrigation treatments within hybrids, drought stress had no effects on ERD of NDF. Finally, and more broadly, the effect of drought stress on NDF degradability of corn for silage is still inconclusive and deserves further investigation. About the latter, the growth of corn plants in a greenhouse does not result in plants with the anatomical structure typically seen in the field.
